# Association between Cardiac Autonomic Function, Oxidative Stress and Inflammatory Response in Impaired Fasting Glucose Subjects: Cross-Sectional Study

**DOI:** 10.1371/journal.pone.0041889

**Published:** 2012-07-31

**Authors:** Ramkumar Thiyagarajan, Senthil Kumar Subramanian, Nishanth Sampath, Madanmohan Trakroo, Pravati Pal, Zachariah Bobby, Sankar Paneerselvam, Ashok Kumar Das

**Affiliations:** 1 Department of Physiology, Jawaharlal Institute of Postgraduate Medical Education and Research, Pondicherry, India; 2 Department of Biochemistry, Jawaharlal Institute of Postgraduate Medical Education and Research, Pondicherry, India; 3 Department of Medicine, Jawaharlal Institute of Postgraduate Medical Education and Research, Pondicherry, India; 4 Advanced Center for Yoga Therapy, Education and Research, Jawaharlal Institute of Postgraduate Medical Education and Research, Pondicherry, India; Scientific Directorate, Bambino Hospital, Italy

## Abstract

**Background:**

The worldwide burden of diabetes in 2030 is projected around 552 million. Diabetes leads to higher risk for cardiovascular diseases (CVD). Altered cardiac autonomic function (CAF) measured by heart rate variability (HRV) is observed in early stages of diabetes but the relationship between impaired fasting glucose (IFG) and HRV is still debatable. The aim of the study was to evaluate the association between CAF, oxidative stress, insulin resistance (IR), and inflammatory response in IFG subjects.

**Subjects and Methods:**

Cross-sectional blinded study. Volunteers recruited from health awareness camps underwent CAF and biochemical tests. Based on fasting plasma glucose (FPG) participants (n = 123) were divided into two groups, normal fasting glucose (n = 76) and IFG (n = 47). The comparison of parameters between the groups was carried out using student *t* test and Mann-Whitney U test for parametric and non-parametric data respectively. The correlation between the parameters was analyzed by Spearman’s rank correlation using SPSS 13.0.

**Results:**

The resting cardiovagal modulation parameters, heart rate response to forced timed breathing, and orthostatic stress were reduced in IFG subjects. Fasting plasma lipid profile, coronary atherogenic lipid risk factors, IR, thiobarbituric acid reactive substance (TBARS), high sensitive C-reactive protein, and tumor necrosis factor alpha were increased and total antioxidant capacity (TAC) was decreased significantly in IFG group but no significant alteration was observed in high-density lipoprotein (HDL-c). Cardiovagal modulation parameters were negatively correlated with triglycerides, FPG, insulin, IR, TBARS, and inflammatory markers and positively with TAC.

**Conclusion:**

There is a continuous interplay between the altered CAF, hyperinsulinemia, IR, oxidative stress parameters, inflammatory response, and IFG in which one factor perpetuates another leading to the progression of disease.

## Introduction

Diabetes, the most common endocrine disorder, is projected to show a worldwide increase from 366 million in the year 2011 to 552 million in the year 2030, out of which, around 101 million is expected to be contributed by India [1]. Diabetic patients have higher risk for cardiovascular diseases (CVDs) which further increases the rate of mortality [2], [3]. Reason for the rate of increase may be lack of observation, follow-up programmes and self-awareness about the conditions of disease. Moreover, the disease manifestations start in the early stages of diabetes and before it gets established as a full blown condition i.e., in the pre-stage called prediabetes. It has been predicted that 25% of the subjects with prediabetes progress to diabetes in 5 years [4].

In 2003, the American Diabetes Association (ADA) defined prediabetes as impaired fasting glucose (IFG) of 100–125 mg/dL (5.6–6.9 mmol/L) or impaired glucose tolerance (IGT) of 140–199 mg/dL (7.8–11.0 mmol/L) after two hours postprandial [5]. Prediabetes is not only associated with increased risk of progression to type-2 diabetes but also with increased CVD risk [4], [6]. Furthermore, Dunstan et al., have demonstrated the presence of microvascular and macrovascular complications in prediabetic state itself [7]. But the degree of progression to type-2 diabetes, intensity of risk factors and cardiovascular complications vary between individuals with IGT and IFG [8]–[10]. In the Baltimore Longitudinal Study on Aging (BLSA), prevalence of IGT and IFG were found to be similar and in addition, progression rates to diabeteswere also similar to IGT, when the lower cut off for diagnosing IFG started from 5.55 mmol/L [11]. According to previous definition of ADA [12], there is adequate evidence to suggest that IGT is strongly associated with CVD morbidity and mortality but the risks predicted through IFG is uncertain and debatable [10], [13]. Recent studies based on new and modified criteria of ADA, lower cut off for IFG was adopted as 5.5 mmol/L showed IFG is also associated with CVD risk [6], [14]. The association between new prediabetes criteria and CVD risk remains to be investigated, since the available data is not sufficient to corroborate it.

Altered cardiac autonomic function (CAF) assessed using heart rate variability (HRV) is associated with metabolic abnormalities including obesity and diabetes [15], [16]. Recent studies showed controversial results with respect to IFG [17] and the results were even insignificant after adding one more cardiovascular risk factor, obesity with it [18]. However, an early diagnosis of altered CAF may lead to an effective management of prediabetic state and reduce the CVD risk. Hence, we planned to elucidate the role of CAF and its association with oxidative stress and inflammatory markers in subjects with IFG.

## Subjects and Methods

### Study Design

This is a cross-sectional blinded study. Volunteers who participated in the study were not aware about which group they belonged to during the study, as the grouping was done after the results of fasting plasma glucose (FPG). The investigators who performed the CAF tests, inflammatory markers, oxidative stress parameters and other biochemical parameters (FPG, insulin and lipid profile) were blinded to each other’s findings.

### Subjects

An approval for awareness programme and the study protocol was obtained from Institute Human ethics committee, Jawaharlal Institute of Postgraduate Medical Education and Research (JIPMER), Pondicherry, India. A total of 414 participants in the age group of 30–60 years from different urban areas of Pondicherry, India, were screened through awareness programmes for diabetes and hypertension conducted by the departments of Physiology and Medicine, JIPMER during the period of Jan 2011 to Oct 2011. Written informed consent was obtained from each participant. 74 participants were excluded from the study due to CVDs, chronic illness, primary autonomic failure, renal insufficiency or any history of medication intake that can affect CAF and FPG. Among the remaining340 participants, only 140 gave written consent to continue their participation in the study. Subsequently, the subjects with fasting plasma glucose (FPG) of more than 125 mg/dL were also excluded (n = 17). A final total of 123 participants were divided into two groups according to the recent ADA 2003 criteria (4);subjects who had FPG in the range of 60–99 mg/dL and 100–125 mg/dL were categorized as NFG group(n = 76; M = 35, F = 41) and IFG group (n = 47; M = 23, F = 24) respectively. We did not exclude subjects with prehypertension (n = 69), out of which 41 subjects were in NFG group and remaining in IFG group.

### Laboratory Measurements

All the participants reported to the lab between 0700 to 0900 hrs. Their personal history like alcohol and smoking habits (number of packs/month), occupational status and medical history were taken. The physical activity of the participants was assessed based on their activity at work, travel to and from places and recreation activities using Global Physical Activity Questionnaire (GPAQ). Physical activity was analyzed by metabolic equivalent (MET), a ratio between work metabolic rate and rest metabolic rate. One MET defined as 1 Kcal/Kg/hour.

#### Anthropometry

Waist circumference was measured with steel, non-elastic tape (make: CESCORF, Brazil) with measurement made mid-way between lower costal border and top of the iliac crest. Subjects’ mean age, gender distribution and waist circumference are mentioned in Table 1.

**Table 1:** Distribution of anthropometric measurements and demographic profile of NFG and IFG group.

Parameters/Group	NFG (n = 76)	IFG (n = 47)
Age (yr)	42.01±7.2	43.55±6.29
Gender distribution	35M–41F	23M–24F
No. of smokers	06 (7.89%)	07 (14.89%)
No. of alcoholics	08 (10.53%)	07 (14.89%)
Family history of diabetes	15 (19.74%)	09 (19.15%)
Family history of hypertension	08 (10.53%)	06 (12.77%)
No. of prehypertensives	41 (53.95%)	28 (60.87%)
Height (cm)	161.36±9.31	160.77±9.48
Weight (kg)	68.89±9.77	69.87±10.82
Waist male (cm)	85.31±8.45	85.52±5.07
Waist female (cm)	85.72±6.68	84.81±4.44

*Data are expressed as mean* ± *SD or frequency; NFG, normal fasting glucose; M, male; F, female; SD, standard deviation; NFG, normal fasting glucose.* *p<*0.05, considered statistically significant.*

#### Cardiac autonomic function tests

After the anthropometric measurements, the subject’s blood pressure (BP) and heart rate (HR) were recorded in comfortable sitting posture. They were explained about the tests of CAF. The tests were performed on subjects with minimal, loose clothing, in a quiet ambient room with dim lighting and room temperature of 24–26°C. CAF tests were done in the following sequence: short-term heart rate variability (HRV) was measured for 5 minutes after comfortable rest in supine position, forced timed breathing (FTB) at the rate of 6 cycles/min for 8 cycles, orthostatic stress (OST) - standing after 20 minutes of supine rest, Valsalva maneuver and isometric handgrip (IHG) at 1/3^rd^ of their maximal strength for the period of 3 minutes. Each procedure was done after comfortable rest period.

The analog signals (lead II ECG and respiration) were converted to digital, using a 16-bit, 16-channel data acquisition system (Biopac MP100, USA) with AcqKnowledge 3.8.2 software. Sampling rate was kept at 500 Hz per channel. Raw ECG data was filtered using band pass filter (2 Hz to 40 Hz). HRV analysis software (version 1.1., Biomedical signal analysis group, University of Kuopio, Finland) which analyzes the frequency spectrum components using fast Fourier transformation and time domain components in the RR trend was used. The results of frequency domain analysis were given as spectral power in ms^2^ including very low frequency (VLF; 0.003 Hz to 0.04 Hz), low frequency (LF; 0.04 Hz to 0.15 Hz) and high frequency (HF; 0.15 Hz to 0.4 Hz). Total spectral power (TP); addition of VLF, LF and HF, LF/HF ratio;ratio of LF power to HF power, low frequency power in normalized units (LFnu)  = LF/(TP–VLF)×100 and similarly HFnu were calculated andthe time domain components include mean and standard deviation of RR intervals (SDNN), mean and standard deviation of HR(SDHR), square root of the mean of the sum of the squares of differences between adjacent RR interval (RMSSD), adjacent RR interval differing more than 50 ms (NN50) and NN50 counts divided by all RR intervals (pNN50). HF, HFnu, SDNN, RMSSD, NN50 and pNN50 reflect cardiovagal tone; LF reflects both sympathetic and parasympathetic activity; VLF component’s physiological explanation is ill defined and it cannot be interpreted based on short-term HRV recordings; LFnu and HFnu represent controlled and balanced activity of sympathetic and parasympathetic nervous system (PSNS) and LF/HF ratio indicates sympathovagal balance.

The reactivity tests included difference between the maximum HR during inspiration and minimum during expiration calculated as HR max–HR min (HR_FTB_), HR response to OST (HR_OST_) calculated as the ratio between longest RR interval around 30^th^ beat and shortest around 15^th^ beat called as 30/15 ratio, HR difference during Valsalva maneuver (HR_VAL_) calculated as max HR during maneuver and minimum HR within 30 seconds after the maneuver and vasoconstrictor reserve (VCR) calculated as the difference between diastolic BP during IHG test and supine diastolic BP in mm Hg. HR_FTB_is an index of cardiac parasympathetic function, HR_OST_ and HR_VAL_ indicate both sympathetic and parasympathetic function and IHG test is a function of sympathetic activity.

#### Biochemical analysis

The FPG was estimated by glucose oxidase-peroxidase method (Genuine Biosystem) and the lipid profile: total cholesterol (TC), triglyceride (TG) and high density lipoprotein cholesterol (HDL-c) were measured by diagnostic kit method (Agappe Diagnostics) using fully automated clinical chemistry analyzer (AU400, Olympus, USA). Low density lipoprotein cholesterol (LDL-c) and very low density lipoprotein cholesterol (VLDL-c) were calculated using Friedwald’s equation. The plasma insulin was measured using direct chemiluminescence immunoassay (Siemens, USA). Insulin resistance (IR) was calculated using plasma fasting glucose and insulin as HOMA-IR i.e., fasting insulin (mU/L) X [fasting glucose (mmol/L)/22.5] [19]. Oxidative stress parameters: TBARS and TAC(Cayman chemical company, USA) and inflammatory markers: hs-CRP (DBC, Canada) and TNF-α (Orgenium, Finland) were measured using ELISA kit according to manufacturer instructions. Coronary atherogenic lipid risk factors like TC/HDL-c, TG/HDL-c, LDL-c/HDL-c, Non-HDL-c and atherogenic index of plasma (AIP): ln(TG)/HDL-c were calculated using fasting lipid profile parameters.

### Statistical Analysis

All analyses were performed with statistical package for social sciences (SPSS) 13.0 for Windows (SPSS, USA). The data were expressed as mean with SD and the categorical data as frequencies. Frequency distributions between the groups were compared with Fischer’s exact test. Natural log transformation was done for extremely variable power spectral components like LF, HF and TP. The comparison of parameters between the groups was carried out using student *t* test for parametric data and Mann-Whitney test for non-parametric data. The association between the parameters was analyzed by Karl Pearson’s correlation or Spearman’s rank correlation. RMSSD and ln(HF) were used for correlation as cardiovagal modulation parameters. A p-value of less than 0.05 was considered statistically significant.

## Results

### Resting Physiological Parameters

HR was found to be significantly higher in IFG group as compared to NFG group. While systolic BP and rate pressure product (RPP) were significantly increased in IFG group, the trend towards increment observed in diastolic BP and mean arterial pressure (MAP) were not significant (Table 2).

**Table 2:** Comparison of resting (sitting) physiological parameters between NFG and IFG group.

Parameters/Group	NFG (n = 76)	IFG (n = 47)	p value
Systolic BP (mmHg)	117.92±10.57	122.49±10.09	0.021
Diastolic BP (mmHg)	79.41±8.11	80.34±8.99	0.563
Heart rate (bpm)	71.96±8.61	75.81±10.61	0.039
MAP (mmHg)	92.25±8.25	94.39±8.61	0.154
RPP (bpm*mmHg/100)	84.92±13.3	92.89±15.07	0.011
Physical activity (MET)	627.95±611.69	387.03±596.6	0.043

*Data are expressed as Mean* ± *standard deviation; p-value* <*0.05 considered as statistically significant.*

*NFG, normal fasting glucose; BP, blood pressure; HR, heart rate; MAP, mean arterial pressure; RPP, rate pressure product; bpm, beats per minute; MET, metabolic equivalent.*

### Cardiac Autonomic Function Test Parameters

When compared to NFG group, the resting cardiovagal modulation parameters, frequency domain indices: ln(HF) (p = 0.002), ln(TP) (p = 0.011), HFnu (p = 0.014) and time domain indices: SDNN (p = 0.023), RMSSD (p = 0.007), NN50 (p = 0.023) and pNN50 (p = 0.021) were significantly reduced in IFG group. Cardiac autonomic reactivity tests: reflex response of HR to FTB (p = 0.076), an important early indicator of diabetic autonomic neuropathy, HR response to Valsalva maneuver (p = 0.399) and BP response to IHG test (p = 0.819) did not differ significantly but HR response to OST assessed by 30∶15 ratio (p = 0.036) differed significantly between IFG and NFG group (Table 3).

**Table 3:** Comparison of cardiac autonomic function test (supine) parameters between NFG and IFG group.

Parameters/Group	NFG (n = 76)	IFG (n = 47)	p value
**Short-term HRV at rest: Frequency domain parameters**			
ln(LF)	2.14±0.4	2.07±0.4	0.317
ln(HF)	2.2±0.45	1.95±0.41	0.002
ln(TP)	2.62±0.34	2.46±0.33	0.011
LF/HF ratio	1.18±1.07	1.98±1.92	0.018
LF nu	47.18±16.47	55.75±19.51	0.014
HF nu	52.82±16.47	44.25±19.51	0.014
**Short-term HRV at rest: Time domain parameters**			
SDNN (ms)	37.82±15.61	30.94±11.92	0.023
RMSSD (ms)	37.59±20.53	27.63±12.95	0.007
NN50 (count)	55.04±56.9	33.96±42.72	0.023
pNN50	16.93±18.37	10.11±12.78	0.021
**Cardiac autonomic reactivity tests**			
HR_FTB_ (bpm)	25.35±9.6	21.74±6.87	0.076
30/15 ratio	1.58±0.23	1.49±0.23	0.036
HR_VAL_ (bpm)	48.13±16.99	47.42±16.33	0.819
VCR (mm Hg)	31.95±10.49	31.6±9.94	0.852

*Data are expressed as Mean ± standard deviation; LF, low frequency spectral power; HF, high frequency spectral power; TP, total spectral power; nu, normalized units; HRV, heart rate variability; RR, R to R wave; SD, standard deviation; RMSSD–root mean square of successive standard deviation of RR interval; NN50, consecutive R to R interval differs more than 50 ms; p, percentage; HR_FTB_, HR difference during forced timed breathing; 30/15 ratio, ratio of longest to shortest RR interval in response to orthostatic stress; HR_VAL_, heart rate difference during and after Valsalva maneuver; VCR, vasoconstrictor reserve. p-value<0.05 considered statistically significant.*

### Biochemical Parameters

Fasting plasma lipid profile parameters such as TC (p = 0.009), TG (p = <0.001), LDL-c (p = 0.044) and VLDL-c (p = <0.001) were significantly higher in IFG group but the HDL-c values were slightly reduced in IFG group as compared to NFG group. Oxidative stress assessed by TBARS (p = 0.02) and TAC (p<0.001), FPG (p<0.001), plasma insulin (p = 0.006) and IR (p<0.001) were higher in IFG group. The inflammatory markers, hs-CRP (p = 0.001) and TNF-α (<0.001) were also significantly increased in IFG group (Table 4).

**Table 4:** Comparison of biochemical parameters between NFG and IFG group.

Parameters/Group	NFG (n = 76)	IFG (n = 47)	p value
FPG (mmol/L)	4.59±0.48	6.1±0.47	<0.001
Total cholesterol (mmol/L)	4.55±0.66	4.89±0.71	0.009
Triglycerides (mmol/L)	1.28±0.46	1.64±0.56	<0.001
HDL-c (mmol/L)	1.01±0.13	0.99±0.08	0.552
LDL-c (mmol/L)	2.95±0.58	3.14±0.56	0.044
VLDL-c (mmol/L)	0.59±0.22	0.76±0.26	<0.001
Insulin (µU/L)	13.39±7.25	15.31±4.78	0.006
IR	2.74±1.53	4.16±1.37	<0.001
TBARS (µmol/L)	3.34±0.72	3.61±0.58	0.020
TAC (µmol/L)	1.88±0.69	1.36±0.45	<0.001
Hs-CRP (µg/mL)	4.18±2.58	5.86±2.67	0.001
TNF-α (pg/mL)	61.33±21.28	90.25±24.62	<0.001

*Data are expressed as Mean* ± *standard deviation; HDL-c, high density lipoprotein cholesterol, LDL-c, low density lipoprotein cholesterol; VLDL-c, very low density lipoprotein cholesterol; IR, insulin resistance; TBARS, thiobarbituric acid reactive substance; TAC, total antioxidant capacity; hs-CRP, high sensitive C-reactive protein; TNF-α-tumor necrosis factor alpha; p-value* <*0.05 considered statistically significant.*

### Atherogenic Lipid Risk Factors

Coronary atherogenic lipid risk factors like TC/HDL-c, TG/HDL-c, LDL-c/HDL-c, Non-HDL-c and AIP of NFG and IFG subjects are represented in Table 5. The TG/HDL-c (p = 0.001), non-HDL-c (p = 0.004) and AIP (p<0.001) were significantly higher in IFG group than NFG group (Table 5).

**Table 5:** Coronary atherogenic lipid risk factors of NFG and IFG group.

Parameters/Group	NFG (n = 76)	IFG (n = 47)	p-value
TC/HDL-c	4.47±0.79	4.74±0.87	0.069
TG/HDL-c	2.95±1.15	3.71±1.36	0.001
LDL-c/HDL-c	2.9±0.91	3.07±0.58	0.234
Non-HDL-c	135.36±25.69	149.14±28.14	0.004
AIP (lnTG/HDL-c)	0.43±0.18	0.54±0.15	<0.001

*Data are expressed as Mean* ± *standard deviation; TC, total cholesterol; HDL-c, high density lipoprotein cholesterol; TG, triglyceride, LDL-c, low density lipoprotein cholesterol; AIP, atherogenic index of plasma. P*<*0.05 considered statistically significant.*

### Correlation between Resting Physiological, Cardiac Autonomic Function Tests and Biochemical Parameters

The resting HR was correlated positively with TG (r = 0.18, p = 0.04), FPG (r = 0.174, p = 0.05), insulin (r = 0.186, p = 0.039), IR (r = 0.248, p = 0.005), hs-CRP (r = 0.275, p = 0.002) and TNF-α (r = 0.174, p = 0.05). Cardiovagal modulation parameters like ln(HF) and RMSSD were negatively correlated with HR, TG, FPG, Insulin, IR, TBARS, hs-CRP and TNF-α and directly with TAC. MAP was positively correlated with TG, FPG, Insulin, IR, TBARS, hs-CRP and TNF-α and negatively with TAC. We observed positive correlation of FPG with HR, sympathetic activity components, inflammatory markers and TBARS. FPG also showed negative correlation with cardiovagal modulation parameters and TAC. Waist circumference, the clinically accepted measure of obesity was significantly correlated with FPG, Insulin, IR, TBARS and TNF-α (Table 6).

**Table 6:** Spearman correlation (‘r’ value) of resting physiological, cardiac autonomic functions with biochemical parameters.

Parameters	HR	TG	FPG	Insulin	IR	TBARS	TAC	hs-CRP	TNF-α
Waist	−0.159	0.137	0.294^#^	0.21*	0.208*	0.197*	−0.121	0.035	0.294^#^
HR	1	0.18*	0.174*	0.186*	0.248^#^	−0.062	−0.123	0.275^#^	0.174*
ln(HF)	−0.321**	−0.209*	−0.308^#^	−0.335**	−0.341**	−0.331**	0.424**	−0.072	−0.308^#^
LF/HF ratio	0.307**	0.179*	0.324**	0.043	0.16	0.052	−0.332**	0.151	0.217^#^
SDNN	−0.292^#^	−0.156	−0.149	−0.339**	−0.329**	−0.295^#^	0.305**	−0.004	−0.149
RMSSD	−0.384**	−0.179*	−0.229*	−0.311**	−0.319**	−0.262^#^	0.417**	−0.033	−0.229*
MAP	0.158	0.352**	0.474**	0.198^#^	0.256^#^	0.424**	−0.466**	0.103	0.474**
VCR	−0.08	0.019	0.155	0.052	0.08	0.024	−0.07	0.182*	0.154
FPG	0.174*	0.426**	1	0.277^#^	0.547**	0.166	−0.391**	0.293**	0.489**
Insulin	0.186*	0.286^#^	0.277^#^	1	0.948**	0.072	−0.527**	0.33**	0.271^#^
IR	0.248^#^	0.379**	0.547**	0.948**	1	0.127	−0.576**	0.39**	0.411**

** p<0.0001, ^#^p<0.005, *p<0.05. *HF, high frequency spectral power; LF/HF ratio: ratio of LF power to HF power; SDNN, standard deviation of R to R interval; RMSSD, root mean square of successive standard deviation of RR interval; MAP, mean arterial pressure; VCR, vasoconstrictor reserve; TG, triglyceride; FPG, fasting plasma glucose; IR, insulin resistance; TBARS, thiobarbituric acid reactive substances; TAC, total antioxidant capacity; hs-CRP, high sensitive C-reactive protein; TNF-α, tumor necrosis factor alpha.*

## Discussion

Resting HR is considered as an index of cardiac autonomic function and predictor of CVDs and mortality [20]. The present study showed a significant escalation of HR in IFG group similar to a population-based study in Taiwan [21]. Elevated HR may indicate sympathetic over activity or decreased cardiovagal tone. The significant rise in LFnu indicating increased sympathetic activity is not substantiated by the insignificant results observed in LF, diastolic BP and IHG test. The rise in LFnu can be relative due to significant fall in HFnu which is supported by significant fall in other cardiovagal modulation parameters like ln(HF), SDNN, RMSSD, NN50 and pNN50. Also significant negative correlation observed between HR and cardiovagal modulation parameters (HF and RMSSD) favors the argument that the increase in HR is primarily due to fall in parasympathetic tone in IFG compared to NFG group and this is consistent with the findings from previous study [21]. Lower HR_FTB_ observed in IFG group compared to NFG group in our study indicates reduced PSNS activity and this goes in hand with previous study of Wu et al [17]. Our findings support the concept that PSNS dysfunction occurs earlier in the course of development of autonomic neuropathy in diabetic subjects [16], [22].

The balance between the two limbs of autonomic nervous system plays an important role in glucose homeostasis [23]. LF/HF ratio is considered as an indicator of sympathovagal balance. LF/HF ratio was positively correlated with fasting glucose and it was higher in IFG group. We observed compensatory increase in fasting plasma insulin level in response to glucose load in IFG group as compared to NFG group [24]. Excess glucose load [25] and insulin [26] can induce ROS production and this in turn can lead to development of IR [27]. Elevated IR observed in IFG group, increases their risk of conversion to diabetes [24]. The findings discussed above explain that there is a continuous interplay between the altered CAF, hyperinsulinemia, IR and glucose load in which one factor perpetuates another leading to the progression of the pathophysiology of metabolic derangement in IFG subjects. In our study, we confirm this with a strong negative correlation of cardiovagal modulation parameters with insulin, IR and FPG.

The increased ROS also damages bilipid cell membrane layer by lipid peroxidation (LPO) [27]. TBARS, a marker of LPO was significantly increased in IFG group. On the other hand, decrease in the antioxidant enzyme activity was observed in IFG as compared to NFG group. Our observations are consistent with the findings of previous studies where oxidative stress was found to be higher in prediabetes and diabetes than in normal subjects [28], [29]. Further we also observed that TBARS correlated indirectly with cardiovagal modulation parameters and directly with FPG, but the exact opposite was observed in the case of TAC.

Inflammatory markers like hs-CRP and TNF-α was significantly increased in IFG group in accordance with previous study [30]. These findings point out that IFG is a state of subclinical inflammation. Inflammatory markers showed a negative correlation with cardiovagal modulation parameters and positive correlation with FPG.

Lipid profile parameters like TC, TG, LDL-c and the derived parameters like TG/HDL-c, non-HDL-c and AIP were significantly increased in IFG group. The increased ROS seen in IFG group would have lead to oxidation of LDL-c, which increases its availability in the circulation. But, the previous studies showed inconsistent results with respect to blood lipid parameters [14], [31]–[33].

The present study exhibited elevated HR, altered CAF in IFG group indicating higher risk for development of CVDs [20], [34]. Elevated IR [24], TC, LDL-c [35] TG [36], ratio of TG to HDL-c [37] which are strongly associated with CVDs were observed in IFG group. Increased oxidative stress and inflammatory response in prediabetic stage predict the development of atherosclerosis and type-2 diabetes [14], [24]. All these observations show that individuals with IFG have higher risk for conversion to diabetes, development of atherosclerosis and cardiovascular diseases in future.

The waist circumference, a clinically accepted tool to assess central obesity did not differ significantly between IFG and NFG group. This may avoid its influence on CAF and metabolic syndrome components [38]. Progression of age also influences CAF [39], [40] and FPG. Though gender has no effect on the prevalence of IFG [8], females as energy conservers are considered to have higher parasympathetic tone than males [40]. Hence, age, gender and waist in both the groups were matched in our study. We observed significantly lower physical activity in IFG than NFG group. Although major confounding factors were minimized, we could not rule out the role of physical activity that might have influenced the derangement of CAF [41], glucose load, IR [42], oxidative stress, inflammatory response and lipid profile [43] in IFG group. Recent studies have explicitly demonstrated the efficacy of lifestyle intervention on preventing type-2 diabetes in subjects with prediabetes [44].

The limitation of this study concerns the use of IFG alone to identify the prediabetic subjects, while the lower cut off for IFG of new criteria has increased the sensitivity comparatively to IGT. We did not measure LDL-c particle size to confirm oxidation of LDL-c. IHG test used to assess sympathetic activity is not a sensitive marker compared to an invasive procedure like muscle sympathetic nerve activity. We did not have beat to beat BP monitor to determine BP variability and baroreflex sensitivity in our subjects. Another limitation that needs to be acknowledged is the sample size of the present study, but it is a preliminary study which was initiated to sensitize the population about diabetic and prediabetic conditions. However, more studies are warranted to delineate the association between CVD risk and prediabetes with large sample size.

In conclusion, the alteration in CAF tests, level of insulin, oxidative stress, inflammatory response, lipid profile and coronary atherogenic lipid risk factors in IFG group indicate their higher risk for conversion to diabetes, development of atherosclerosis and cardiovascular diseases in future. Short-term heart rate variability analysis, a noninvasive measurement for cardiac autonomic function, can be used along with blood glucose measurement in diabetics and prediabetics to assess cardiovascular risk. It is advisable to introduce standard lifestyle modifications [45] right from the stage of prediabetes for delaying the progression of prediabetes to diabetes and to reduce the incidence of CVD. This may obviate the need for expensive and complicated therapies.
